# Heckman imputation models for binary or continuous MNAR outcomes and MAR predictors

**DOI:** 10.1186/s12874-018-0547-1

**Published:** 2018-08-31

**Authors:** Jacques-Emmanuel Galimard, Sylvie Chevret, Emmanuel Curis, Matthieu Resche-Rigon

**Affiliations:** 1 INSERM U1153, Epidemiology and Biostatistics Sorbonne Paris Cité Research Center (CRESS), ECSTRA team, Service de Biostatistique et Information Médicale, Hôpital Saint-Louis, AP-HP, 1 avenue Claude Vellefaux, Paris, F-75010 France; 2 Paris Diderot University – Paris 7, Sorbonne Paris Cité, Paris, F-75010 France; 3 Service de Biostatistique et Information Médicale, Hôpital Saint-Louis, Paris, F-75010 France; 4 INSERM UMR-S 1144, Équipe 1, Université Paris Descartes, Université Paris Diderot, Sorbonne Paris Cité, Paris, F-75013 France; 5 Laboratoire de biomathématiques – plateau iB2, faculté de pharmacie, Université Paris Descartes, Sorbonne Paris Cité, Paris, F-75006 France

**Keywords:** Heckman’s model, Missing data, Missing not at random (MNAR), Multiple imputation by chained equation (MICE), Sample selection method

## Abstract

**Background:**

Multiple imputation by chained equations (MICE) requires specifying a suitable conditional imputation model for each incomplete variable and then iteratively imputes the missing values. In the presence of missing not at random (MNAR) outcomes, valid statistical inference often requires joint models for missing observations and their indicators of missingness. In this study, we derived an imputation model for missing binary data with MNAR mechanism from Heckman’s model using a one-step maximum likelihood estimator. We applied this approach to improve a previously developed approach for MNAR continuous outcomes using Heckman’s model and a two-step estimator. These models allow us to use a MICE process and can thus also handle missing at random (MAR) predictors in the same MICE process.

**Methods:**

We simulated 1000 datasets of 500 cases. We generated the following missing data mechanisms on 30% of the outcomes: MAR mechanism, weak MNAR mechanism, and strong MNAR mechanism. We then resimulated the first three cases and added an additional 30% of MAR data on a predictor, resulting in 50% of complete cases. We evaluated and compared the performance of the developed approach to that of a complete case approach and classical Heckman’s model estimates.

**Results:**

With MNAR outcomes, only methods using Heckman’s model were unbiased, and with a MAR predictor, the developed imputation approach outperformed all the other approaches.

**Conclusions:**

In the presence of MAR predictors, we proposed a simple approach to address MNAR binary or continuous outcomes under a Heckman assumption in a MICE procedure.

**Electronic supplementary material:**

The online version of this article (10.1186/s12874-018-0547-1) contains supplementary material, which is available to authorized users.

## Background

In clinical epidemiology, missing data are generally classified as (i) missing completely at random (MCAR); (ii) missing at random (MAR) when, conditional on the observed data, the probability of data being missing does not depend on unobserved data; or (iii) missing not at random (MNAR) when, conditional on the observed data, the probability of data being missing still depends on unobserved data, i.e., neither MCAR nor MAR [1, 2]. Unfortunately, the missing data mechanisms of MNAR, MAR and MCAR are generally not testable unless there are direct modelisations of the missing data mechanisms. Although methods for handling MCAR or MAR data in clinical epidemiology have been widely described and studied, methods adapted for MNAR mechanisms are less studied.

In the presence of MNAR missing outcomes, valid statistical inference implies describing the missing data mechanism [1, 3]. Hence, it often requires joint models for missing outcomes and their indicators of missingness [4]. Two principal factorisations of these joint models have been proposed: pattern-mixture models and selection models [1, 5–7]. The first consists of using different distributions to model individuals with and without missing observations [8, 9]. The second directly models the relationship between the risk of a variable being missing and its unseen value. It involves defining an analysis model for the outcome and a selection model (i.e. the missing data mechanism). It generally relies on a bivariate distribution to model the outcome and its missing binary indicator simultaneously [10]. This approach, called sample selection model, Tobit type-2 model [11] or Heckman’s model, was first introduced by Heckman for continuous outcomes [12, 13]. For continuous outcomes, two approaches have been proposed to estimate the model parameters: a one-step process that directly estimates all parameters of the joint model using the maximum likelihood estimator [11] and a two-step process [12, 13]. The first step of the latter consists of estimating the parameters of the selection model. The second step consists of fitting the outcome model adjusted on a correction term named “inverse Mills ratio” (*IMR*), which is obtained via the first step. *IMR* corresponds to the mean of the conditional distribution of the outcome within the bivariate normal distribution knowing that the outcome has been observed [14]. This allows unbiased estimates of the parameters of the outcome model to be calculated.

For binary outcomes, sample selection methods rely on a different model. This model is not simply an adaptation of the continuous case and notably is not simply an adaptation of the two-step estimator with a different outcome model as a generalised linear model. In the setting of binary outcomes, the use of a bivariate probit model and a one-step maximum likelihood estimator is mandatory [10]. Indeed, the use of a Heckman’s model implies linking the outcome model and the selection model by their error terms. Some authors, through analogy with Heckman’s two-step estimator, proposed modelling binary outcomes using a probit model adjusted on the *IMR* [15]. Despite the misuse of such approaches, it has been specifically demonstrated that the use of a two-step approach including the *IMR* in a probit model for binary outcomes is not valid [10, 16]. More generally, Heckman’s two-step estimator could not be extended straightforwardly to general linear outcome models by plugging *IMR* into the linear predictor. It relies on the fact that outcome expectation in non-linear models subject to selection does not involve a simple corrector term in the linear predictor [16].

If Heckman’s model handles MNAR missing binary outcomes well using a bivariate probit model, then in the presence of additional missing data on predictors, there is no process that can address all the missing data simultaneously. In this setting, missing data on predictors are typically treated using a non-satisfactory *complete-predictors* approach, i.e., cases with at least one missing predictor are removed from the analysis. In the presence of missing data on more than one variable (including the outcome), multiple imputation (MI) appears to be one of the most flexible and easiest method to apply due to the numerous types of variables handled and the extensive development of statistical packages dedicated to its implementation [17]. Galimard et al. [18] previously developed an approach based on a conditional imputation model for an MNAR mechanism using a Heckman’s model and a two-step estimator to impute MNAR missing continuous outcomes. This approach allows imputing MAR missing covariates and MNAR missing outcomes within a multiple imputation by chained equations (MICE) procedure [18]. MICE specifies a suitable conditional imputation model for each incomplete variable and iteratively imputes the missing values until convergence. The key concept of MI procedures is to use the distribution of the observed data to draw a set of plausible values for the missing data. Thus, imputing missing MNAR binary outcomes implies developing valid methods to obtain a valid distribution of missing binary outcomes. As mentioned above, the direct extension of the work of Galimard et al. [18] on continuous outcomes cannot be considered because it involves a two-step estimator which is not compatible with Heckman’s model with binary outcomes.

### Aims of this work

The first aim of this work is to propose an approach to handle MNAR binary outcomes. To our knowledge, the use of sample selection models as imputation models has never been proposed for missing binary outcomes, which is a current framework in clinical research. Thus, we propose developing an imputation method for binary outcomes based on a bivariate probit model associated with a one-step maximum likelihood estimator.

The second aim is to extend this approach for continuous outcomes proposing a new approach for the issue raised by Galimard et al. [18]. Indeed, for continuous outcomes, one of the main drawbacks of Heckman’s two-step estimator is that the uncertainties of the first step estimates are not taken into account in the second step. Indeed, *IMR* is considered as known observed values in the second step, whereas they have been estimated in the first step. Thus, the uncertainties around the final estimates are not fully assessed using a two-step estimator [19]. This point could impact the quality of the imputation. This is the reason why we hypothesised that the use of a one-step estimator could also improve the performance of Heckman’s model as an imputation model for continuous outcomes. Therefore, we also proposed a new approach for continuous missing outcomes.

The final aim is to integrate the current developed MNAR model into a MICE procedure. It will handle both MNAR outcomes and MAR predictors in the same process.

In what follows, we introduce the study that motivated this work. Then, the “Methods” section section develops our proposed imputation model using one-step ML estimation for binary and continuous outcomes. The “Results” section section presents the evaluation of its performance using a simulation study and an illustrative example using data from our motivating example. Finally, a discussion and some conclusions are provided.

## Motivating example: the BIVIR study

The BIVIR study was a three-arm, parallel, randomised clinical trial that aimed to assess the efficacy of the Oseltamivir-Zanamivir combination relative to each monotherapy in patients with seasonal influenza. This study was conducted by 145 general practitioners throughout France during the 2008-2009 seasonal influenza epidemic and included 541 patients. Primary analyses of the trial showed that the Oseltamivir-Zanamivir combination is less effective than Oseltamivir monotherapy and not significantly more effective than Zanamivir monotherapy based on the proportion of patients with nasal influenza reverse transcription (RT)-PCR below 200 copies genome equivalent (cgeq)/ *μ*l at day 2 after randomisation [20]. We focused our work on evaluating the impact of the treatment group on adherence adjusted on the first day severity score of flu symptoms. Adherence was defined as completing the full treatment between day 1 and day 5 and was self-reported by the patient. Unfortunately, adherence was missing for 115 (21%) patients. It was reasonable to suspect that patients who decided to stop treatment might be more likely to not record data on their adherence, resulting in an MNAR mechanism. The severity score corresponding to flu symptoms was measured as a weighted sum (ranging from 0 to 78) of 13 intensity symptoms [21]. The score was missing for 114 (21%) patients, and a MAR mechanism was suspected.

## Methods

### Heckman’s model

Let *Y*_*i*_ be a binary outcome and *X*_*i*_ be a *p*-vector of covariates for individual *i*=1,...,*n*. Adopt the following probit regression model as the outcome model:

1 $$ P(Y_{i}=1|X_{i})= \Phi(X_{i}\beta)   $$

where *Φ* is the standard normal cumulative distribution function and *β* is a *p*-vector of fixed effects. Assuming an underlying MNAR mechanism for *Y*, introduce a selection model that represents the non-random sampling of the missingness process:

2 $$ P\left(R_{yi}=1|X^{s}_{i}\right)=\Phi\left(X^{s}_{i}\beta^{s}\right)   $$

where *R*_*yi*_ is an indicator of *Y*_*i*_ missingness (equal to 1 if *Y*_*i*_ is observed and 0 if *Y*_*i*_ is missing), $X_{i}^{s}$ is a *q*-vector of observed covariates potentially associated with the missingness mechanism, and *β*^*s*^ is an unknown *q*-vector of coefficients.

According to the bivariate probit model, define *Y*^′^ and *Ry*′ as two latent normally distributed variables associated with *Y* and *R*_*y*_, respectively, such that for individual *i*, *Y*_*i*_=1 if *Yi*′>0 and *Y*_*i*_=0 otherwise and *R*_*yi*_=1 if *Ryi*′>0 and *R*_*yi*_=0 otherwise. Heckman’s model considers that the two latent formulations of the selection and outcome models are linked through their error terms, which follow a bivariate normal distribution. The joint model of the outcome and selection models is defined as:

3 $$ {\begin{aligned} \begin{array}{ll} R_{yi}'&=X^{s}_{i}\beta^{s} +\varepsilon_{i}^{s} \\ Y'_{i}&= X_{i}\beta + \varepsilon_{i} \end{array},~~ \text{with}~~ \left(\begin{array}{ll} \varepsilon^{s} \\ \varepsilon \end{array} \right) \sim N\left(\left(\begin{array}{ll} 0 \\ 0 \end{array} \right) \text{,} \left(\begin{array}{cc} 1 & \rho\\ \rho & 1 \end{array} \right)\right)  \end{aligned}}  $$

where *ρ* corresponds to the correlation coefficient between the error terms of the selection model $\left (\varepsilon ^{s}_{i}\right)$ and outcome model (*ε*_*i*_). When *ρ* equals 0, the selection and outcome models are independent, $E\left (R_{yi}'|Y_{i},X_{i},X_{i}^{s}\right)$ does not depend on *Y*_*i*_, and the mechanism is MAR. When *ρ* is not equal to 0, $E\left (R_{yi}'|Y_{i},X_{i},X_{i}^{s}\right)$ depends on *Y*_*i*_, and the mechanism is MNAR. The larger *ρ* is, the stronger the MNAR mechanism is.

For a continuous outcome, Heckman’s model given in Eq. (3) is simplified as *Y*_*i*_, the non-latent outcome instead of *Yi*′, is directly inserted in the joined model. The joint model for continuous outcomes is presented below:

4 $$ {\begin{aligned} \begin{array}{ll} R_{yi}'&=X^{s}_{i}\beta^{s} +\varepsilon_{i}^{s} \\ Y_{i}&= X_{i}\beta + \varepsilon_{i} \end{array},~~ \text{with}~~ \left(\begin{array}{ll} \varepsilon^{s} \\ \varepsilon \end{array} \right) \sim N\left(\left(\begin{array}{ll} 0 \\ 0 \end{array} \right) \text{,} \left(\begin{array}{cc} 1 & \rho\sigma_{\varepsilon}\\ \rho\sigma_{\varepsilon} & \sigma_{\varepsilon} \end{array} \right)\right)  \end{aligned}}  $$

where *σ*_*ε*_ is the variance of error terms (*ε*_*i*_).

### Model estimation

#### Maximum likelihood estimator

The parameters of the Heckman’s model (*β*,*β*^*S*^,*ρ*) are directly obtained by maximising the following log-likelihood of the joint bivariate probit model [10, 15, 19]:

$${\begin{aligned} l &= \sum_{\{i:R_{y}=0\}} \log \Phi\left(-X^{s}_{i}\beta^{s}\right) \\ & \quad + \sum_{\{i:R_{y}=1,Y_{i}=1\}} \log\Phi_{2}\left(X_{i}\beta,X_{i}^{s}\beta^{s},\rho\right)\\ &\quad + \sum_{\{i:R_{y}=1,Y_{i}=0\}} \log\Phi_{2}\left(-X_{i}\beta,X_{i}^{s}\beta^{s},-\rho\right) \end{aligned}} $$

where *Φ*_2_ corresponds to the binormal cumulative density function.

For a continuous outcome, the one-step estimator consists of estimating the parameters of the joint model (*β*,*β*^*S*^,*ρ*,*σ*_*ε*_) via the following log-likelihood [14]:

$${\begin{aligned} l &=\sum_{\{i:R_{y}=0\}} \log \Phi\left(-X^{s}_{i}\beta^{s}\right) \\ & \quad +\sum_{\{i:R_{y}=1\}} \left[ \log \Phi\left(\frac{X^{s}_{i}\beta^{s}+\frac{\rho}{\sigma_{\varepsilon}} (Y_{i}-X_{i}\beta)}{\sqrt{1-\rho^{2}}}\right) \right.\\ & \left.\quad -\frac{1}{2}\log2\pi-\log\sigma_{\varepsilon}-\frac{1}{2}\frac{(Y_{i}-X_{i}\beta)^{2}}{\sigma_{\varepsilon}^{2}}{\vphantom{\log \Phi\left(\frac{X^{s}_{i}\beta^{s}+\frac{\rho}{\sigma_{\varepsilon}} (Y_{i}-X_{i}\beta)}{\sqrt{1-\rho^{2}}}\right)}}\right]  \end{aligned}} $$

#### Two-step estimator

For a continuous outcome, Heckman proposed a two-step approach to estimate the parameters of the joint model given in Eq. (4). His development comes from the expression of the following conditional expectation of the outcome [10]:

5 $$ E(Y_{i}|X_{i},X^{s}_{i},R_{yi}=1)=X_{i}\beta +\rho\sigma_{\varepsilon}\lambda_{i}   $$

where $\lambda _{i}=\phi \left (X^{s}_{i}\beta ^{s}\right)/\Phi \left (X^{s}_{i}\beta ^{s}\right)$ is called the “inverse Mills ratio” (*IMR*); *ϕ* corresponds to the probability density function of the normal distribution. As the *IMR* of each individual corresponds to an error term resulting from the probit selection model [22], Heckman proposed the following two-step procedure:

1. Estimate selection model parameters $\left (\widehat {\beta ^{S}}\right)$ by maximum likelihood
2. For each observed *i*, compute $\widehat {\lambda _{i}}$ using $\widehat {\beta ^{S}}$
3. Estimate $\widehat {\beta }$ from Eq. (5)

#### Exclusion-restriction rule

In practice, Heckman’s model must avoid collinearity between the two linear predictors of the outcome model and the selection model. Indeed, if the variables included in the selection and outcome models are exactly the same, then *E*[ *Y*_*i*_|*X*_*i*_,*R*_*yi*_=1]=*X*_*i*_*β*+*ρσ*_*ε*_*λ*_*i*_ is only identified through the *IMR* (*λ*) producing collinearity issues and possibly erroneous estimation. To avoid this concern, it has been recommended to include at least a supplementary variable in the selection equation [14, 22, 23]. Ideally, this supplementary variable should be linked to the indicator of missingness and linked to the outcome [24].

### Imputation model using Heckman’s model

Under the MAR mechanism, imputation approaches use the conditional distribution of observed *Y* given the other covariates to impute the missing *Y*. However, in Heckman’s model, the conditional expectations of the observed and missing *Y* are different. For a binary outcome ([10], p. 921):

6 $$ P\left(Y_{i}=1|X_{i},X_{i}^{s},R_{yi}=0\right)=\frac{\Phi_{2}\left(X_{i}\beta,-X_{i}^{s} \beta^{s},-\rho\right)}{\Phi\left(- X_{i}^{s}\beta^{s}\right)}   $$

We propose using Eq. (6) to define the imputation model for binary outcomes.

#### Imputation algorithm

For a binary outcome, consider Heckman’s model parameters *θ*=(*β*,*β*^*s*^,*ρ*). The imputation algorithm consists of the following steps:

1. Use the one-step estimator to obtain Heckman’s model parameters $\left (\widehat {\theta },\widehat {\Psi }\right)$ where $\widehat {\Psi }$ is the variance-covariance matrix of $\widehat {\theta }$
2. Draw *θ*^∗^ from $N\left (\widehat {\theta }\text {,} \widehat {\Psi }\right)$
3. Draw $Y^{*}_{i}$ from a Bernoulli distribution with parameter $p^{*}_{i}$ from:
  $$p^{*}_{i}=\frac{\Phi_{2}\left(X_{i}\beta^{*},-X^{s}_{i} \beta^{s*},-\rho^{*}\right)}{\Phi\left(- X^{s}_{i} \beta^{s*}\right)}$$

For a continuous outcome, Eq. (6) becomes ([10], p. 913):

7 $$ E\left(Y_{i}|X_{i},X_{i}^{s},R_{yi}=0\right)= X_{i}\beta+\rho\sigma_{\varepsilon}\frac{-\phi\left(X^{s}_{i}\beta^{s}\right)}{\Phi\left(-X^{s}_{i}\beta^{s}\right)}   $$

With model parameters *θ*=(*β*,*β*^*s*^,*σ*_*ε*_,*ρ*), in the third step of the *imputation algorithm*:

3 Draw *Y*^∗^ from:
  $$Y^{*}_{i}=X_{i} \beta^{*} + \rho^{*} \sigma_{\varepsilon}^{*}\frac{-\phi\left(X^{s}_{i} \beta^{s*}\right)}{\Phi\left(-X^{s}_{i}\beta^{s*} \right)} + \varepsilon^{*} ~~\text{with}~~\varepsilon^{*} \sim N\left(0,~\sigma_{\varepsilon}^{*2}\right) $$

### Multiple imputation by chained equations using Heckman’s imputation model

The final aim of this work is to provide a global framework to impute MNAR outcomes and MAR predictors through a MICE procedure. This procedure requires specifying conditional imputation models for each variable with missing data. The global procedure starts with an initial fill of all missing data using random draws from observed values. The posterior predictive distribution of the first incomplete variable is obtained using all observed values. Then, for a given observation with a missing value of the first variable, imputations are generated given all the other variables. Following variables with missing values are similarly repeatedly imputed in an iterated sequence. The key point of chained equation is that consecutive iterations use imputed values of the previous. Then missing value are iteratively imputed until convergence (at least 10 cycles) [17]. The theoretical properties of MICE are not well understood: except in simple cases, conditional imputation models do not correspond to any joint model [25, 26]. However, it performs well in practice [27, 28]. This procedure is realised in parallel to obtain several imputed datasets. Analyses and Rubin’s rules are then applied to obtain the final estimations of the parameters of interest.

We propose using Heckman’s imputation model for MNAR outcomes and standard imputation regression models for missing predictors, such as linear models for continuous covariates and logistic models for binary covariates. In this framework, Galimard et al. [18] proved that the missing data indicator of MNAR outcomes should be included in the imputation models of all other variables. The MICE algorithm involves defining conditional imputation models. In our case the definition of such imputation models will depend on the type of the missing mechanism:

- Heckman’s imputation model for MNAR outcome, specifying outcome and selection models
- General linear imputation models for MAR predictors as described by van Buuren et al. [2] adding *R*_*y*_ and the outcome to other variables in the linear predictors

## Simulation study

### Data-generating process

We generated three normally independent and identically distributed variables, *X*_1_, *X*_2_ and *X*_3_, with *X*_*j*_∼*N*(0,*σ*^2^). Two error terms, *ε* and *ε*^*s*^, were generated using *ρ* fixed at 0, 0.3 and 0.6 to simulate MAR, light MNAR and heavy MNAR settings from a bivariate normal distribution according to the model given in Eq. (3).

For binary outcomes, *Y* was generated as follows: if *β*_0_+*β*_1_*X*_1_+*β*_2_*X*_2_+*ε*>0, then *Y*=1; otherwise, *Y*=0. The missing indicator *R*_*y*_ of *Y* was generated according to the following algorithm: if $\beta _{0}^{s}+\beta _{1}^{s} X_{1}+\beta _{2}^{s} X_{2}+\beta _{3}^{s} X_{3}+\varepsilon ^{s}>0$, then *R*_*y*_=1; otherwise, *R*_*y*_=0.

For continuous outcomes, *Y* was generated according to *Y*=*β*_0_+*β*_1_*X*_1_+*β*_2_*X*_2_+*ε*. Note that in that case and according to the model given in Eq. (4), *σ*_*ε*_=1.

We fixed *σ*^2^ to 0.5 and (*β*_0_,*β*_1_,*β*_2_) to (0,1,1). $\left (\beta _{0}^{s},\beta _{1}^{s},\beta _{2}^{s},\beta _{3}^{s}\right)$ were fixed to (0.75,1,-0.5,1), which resulted in approximately 30% missing data for the outcome.

To evaluate the robustness of our approach, we also generated a non-Heckman MNAR mechanism by directly including *Y* in the following selection equation: $P(R_{y}=1)= logit\left (\beta _{0}^{sl}+X_{1}-0.5 \times X_{2}+X_{3}+\beta _{Y}^{sl} Y\right)$. Two sets of parameters were considered. To obtain approximately 30% missing data on *Y*, we fixed $\beta _{0}^{sl}$ to 0.60 and 0.20 for binary outcomes and to 1.31 and 1.86 for continuous outcomes, with $\beta _{Y}^{sl}$ equal to 0, 1 and 2.

We first simulated scenarios with only missing outcomes to validate our approach in a simple setting. Then, to evaluate the performance of the MICE process, we generated missing data on *X*_2_ using two MAR mechanisms depending on either (*X*_1_,*Y*) or (*X*_1_,*X*_3_). Thus, *R*_2_, the indicator of *X*_2_ missingness, was defined by either:

- *P*(*R*_2_=1|*X*_1_,*X*_3_)=*Φ*(0.25+*X*_1_+*X*_3_)
- $P(R_{2}=1|X_{1},Y)=\Phi \left (\beta _{0}^{R_{2}}+X_{1}+Y\right)$

$\beta _{0}^{R_{2}}$ was fixed to 1.10 and 0.25 for binary and continuous outcomes, respectively. We obtained approximately 30% missing data for *X*_2_.

A total of *N*=1000 independent datasets of size 500 were generated for each setting. The sample size was chosen to be similar to our motivating example.

### Analysis methods

The analysis models were probit models and linear models for binary and continuous outcomes, respectively, including *X*_1_ and *X*_2_ as predictors. The simulated data were first analysed prior to data deletion as a benchmark. The incomplete data were then analysed using the following methods:

- Complete case analysis (*CCA*).
- Heckman’s model (*HEml*) consisting of one-step ML estimation, as described in the “Methods” section for binary and continuous outcomes.
- Multiple imputation using Heckman’s one-step ML estimation (*MIHEml*), as described in the “Methods” section.

For continuous outcomes exclusively, two-step approaches have also been performed.

- Heckman’s two-step estimation (*HE*2*steps*) consisting of Heckman’s two-step estimator for continuous outcomes as described in the “Methods” section for continuous outcomes.
- Multiple imputation using Heckman’s two-step model estimation (*MIHE*2*steps*) for continuous outcomes, as described in Galimard et al. [18].

For *HEml*, *MIHEml*, *HE*2*steps*, and *MIHE*2*steps*, the selection equation included *X*_1_, *X*_2_ and *X*_3_. For *MIHEml* and *MIHE*2*steps*, the incomplete data were imputed *m*=50 times, and final estimates were obtained by applying Rubin’s rules for small samples [29].

For scenarios with missing *X*_2_: (1) for the *HEml* and *HE*2*steps* approaches, observations with missing *X*_2_ were deleted from the analyses as previously described in the *complete-predictors* approach; (2) for *MIHEml* and *MIHE*2*steps*, a MICE procedure was applied. *X*_2_ was imputed using a linear regression model and an approximate proper imputation algorithm [2]. As recommended, we included *R*_*y*_ and *Y* in its imputation model [2, 18]. Twenty iterations of the chained equation process were applied.

In each data-generating scenario, the performance of each method was assessed by computing the percent relative bias (*%Rbias*), the root mean square of the estimated standard error (*SE*_*cal*_), the empirical Monte Carlo standard error (*SE*_*emp*_), the root mean square error (*RMSE*) and the percent of the coverage of nominal 95% confidence intervals (Cover) of *β*_1_ and *β*_2_.

### Computational settings

Simulations and analyses were performed using R statistical software, version 3.3.0 [30]. We computed the imputation procedure within the *mice* R package version 2.25 [31]. Heckman’s One-step model estimator was supplied by functions *semiParBIV()* and *copulaSampleSel()* of the *GJRM* R package version 0.1-1, for binary and continuous cases respectively [19, 32]. Our code is available in the supplementary materials (S1 for binary outcomes and S2 for continuous outcomes). Heckman’s two-step model estimator was performed using the function *heckit()* of package *sampleSelection* version 1.0-4 [14].

## Results

In this section, only the results of *β*_1_ estimations are presented. *β*_2_ estimations are presented in Additional file 1.

### Only missing data on outcome *Y*

**Fig. 1:**
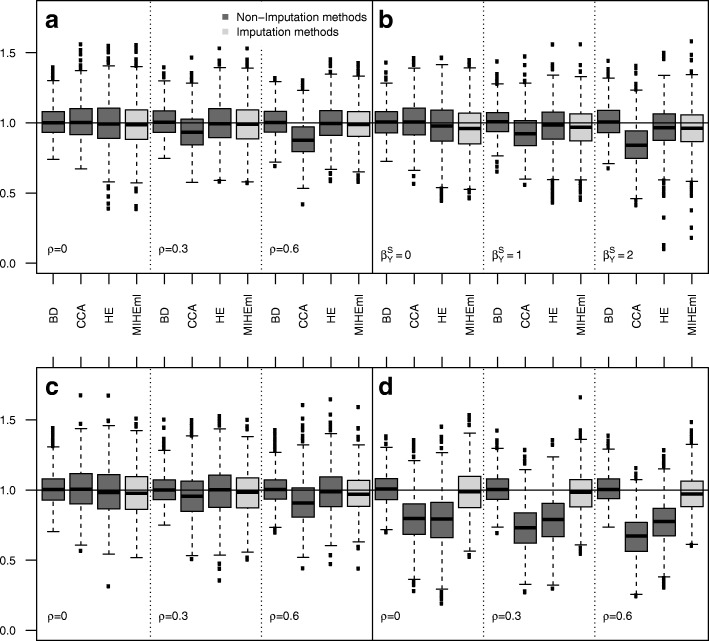
Binary outcome Boxplot of *β*_1_ estimates on the 1000 simulations associated to Table 1 (plot **a**), Table 3 (plot **b**), Table 5 left (plot **c**) and Table 5 right (plot **d**)

Table 1 (Fig. 1) presents the results of the simulation study based on a scenario with missing binary outcome *Y* and complete predictors *X*. When *Y* is missing due to a MAR mechanism (*ρ*=0), all methods provide unbiased estimates of *β*_1_ (relative biases less than 2%). The standard errors of the approaches using Heckman’s model are greater than those of *CCA*. Nevertheless, all coverages are close to their nominal values. In the presence of an MNAR mechanism, *CCA* is biased 6.1% with *ρ*=0.3 and 11.9% with *ρ*=0.6. *HEml* and *MIHEml* are unbiased. The results for *β*_2_ are similar (Additional file 1: Table S8).

**Table 1:** Binary *Y*: Simulation results for *β*_1_=1 with *ρ*=0, representing a MAR mechanism, and *ρ*=0.3 and 0.6, representing an MNAR mechanism

Methods	*ρ*	*%* *R* *b* *i* *a* *s*	*S* *E*_*cal*_	*S* *E*_*emp*_	RMSE	Cover
Before	0	0.7	0.108	0.109	0.109	94.9
deletion	0.3	1.1	0.109	0.109	0.110	95.9
	0.6	0.9	0.109	0.109	0.109	95.2
CCA	0	1.2	0.137	0.137	0.137	95.4
	0.3	-6.1	0.135	0.135	0.148	92.0
	0.6	-11.9	0.135	0.134	0.179	83.5
HEml	0	-0.3	0.161	0.163	0.163	95.0
	0.3	-0.1	0.148	0.151	0.150	94.8
	0.6	-0.1	0.134	0.132	0.132	96.1
MIHEml	0	-1.0	0.159	0.161	0.162	94.2
	0.3	-1.0	0.148	0.150	0.150	95.5
	0.6	-0.9	0.135	0.132	0.133	95.4

*%Rbias*: % relative bias; *SE*_*cal*_: Root mean square of the estimated standard error; *SE*_*emp*_: Empirical Monte Carlo standard error; RMSE: Root mean square error; Cover: % coverage of the nominal 95% confidence interval; CCA: Complete case analysis; HEml: Heckman’s one-step ML estimation; MIHEml: Multiple imputation using Heckman’s one-step ML estimation

**Fig. 2:**
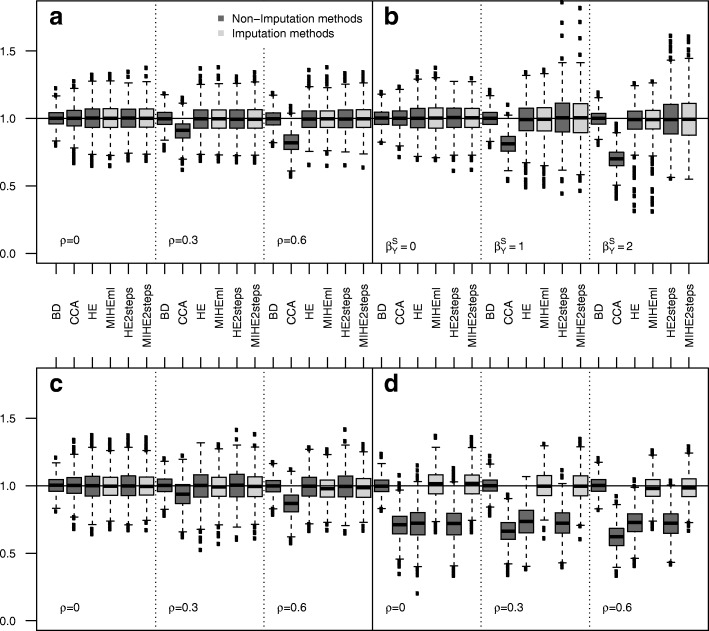
Continuous outcome Boxplot of *β*_1_ estimates on the 1000 simulations associated to Table 2 (plot **a**), Table 4 (plot **b**), Table 6 left (plot **c**) and Table 6 right (plot **d**)

The results of the simulations that considered missing data on a continuous outcome are presented in Table 2 (Fig. 2). Compared to a binary outcome, similar results are observed. *HEml*, *HE*2*steps*, *MIHEml* and *MIHE*2*steps* presented similar results concerning biases; nevertheless, the standard errors obtained with *HEml* and *MIHEml* with *ρ*≠0 are smaller than those observed with *HE*2*steps* and *MIHE*2*steps*, while the confidence intervals remain near 95%. The results for *β*_2_ are similar (Additional file 1: Table S9).

**Table 2:** Continuous *Y*: Simulation results for *β*_1_=1 with *ρ*=0, representing a MAR mechanism, and *ρ*=0.3 and 0.6, representing an MNAR mechanism

Methods	*ρ*	*%* *R* *b* *i* *a* *s*	*S* *E*_*cal*_	*S* *E*_*emp*_	RMSE	Cover
Before	0	0.0	0.064	0.064	0.064	95.1
deletion	0.3	0.0	0.063	0.065	0.065	95.0
	0.6	-0.2	0.064	0.064	0.064	94.3
CCA	0	0.1	0.083	0.084	0.084	95.1
	0.3	-9.1	0.082	0.081	0.122	80.3
	0.6	-17.8	0.078	0.079	0.194	38.2
HEml	0	0.0	0.103	0.103	0.103	95.2
	0.3	-0.4	0.101	0.101	0.101	94.6
	0.6	-0.4	0.092	0.092	0.092	94.2
MIHEml	0	0.0	0.105	0.103	0.103	94.7
	0.3	-0.3	0.103	0.102	0.102	95.3
	0.6	-0.3	0.096	0.094	0.094	94.8
HE2steps	0	0.0	0.103	0.102	0.102	95.4
	0.3	-0.4	0.103	0.103	0.103	94.6
	0.6	-0.2	0.100	0.099	0.099	95.4
MIHE2steps	0	0.0	0.105	0.103	0.103	95.2
	0.3	-0.4	0.104	0.104	0.104	94.0
	0.6	-0.2	0.103	0.100	0.099	95.2

*%Rbias*: % relative bias; *SE*_*cal*_: Root mean square of the estimated standard error; *SE*_*emp*_: Empirical Monte Carlo standard error; RMSE: Root mean square error; Cover: % coverage of the nominal 95% confidence interval; CCA: Complete case analysis; HEml: Heckman one-step ML estimation; MIHEml: Multiple imputation using Heckman’s one-step ML estimation; HE2steps: Heckman’s two-step estimation; MIHE2steps: Multiple imputation using Heckman’s two-step estimation

**Table 3:** Binary *Y* and logit selection model: Simulation results for *β*_1_=1 estimates

Methods	$\beta _{Y}^{sl}$	*%* *R* *b* *i* *a* *s*	*S* *E*_*cal*_	*S* *E*_*emp*_	RMSE	Cover
Before	0	0.9	0.109	0.110	0.110	95.3
deletion	1	1.0	0.109	0.103	0.103	96.4
	2	1.2	0.109	0.115	0.115	94.4
CCA	0	1.5	0.135	0.137	0.138	95.8
	1	-7.2	0.133	0.131	0.149	90.6
	2	-15.4	0.134	0.146	0.212	74.0
Heml	0	-2.4	0.167	0.170	0.171	93.5
	1	-2.5	0.153	0.152	0.154	96.0
	2	-3.5	0.144	0.152	0.156	94.9
MIHEml	0	-4.0	0.163	0.163	0.168	93.9
	1	-3.7	0.152	0.152	0.156	95.2
	1	-4.2	0.145	0.155	0.160	94.9

*%Rbias*: % relative bias; *SE*_*cal*_: Root mean square of the estimated standard error; *SE*_*emp*_: Empirical Monte Carlo standard error; RMSE: Root mean square error; Cover: % coverage of the nominal 95% confidence interval; CCA: Complete case analysis; HEml: Heckman’s one-step ML estimation; MIHEml: Multiple imputation using Heckman’s one-step ML estimation

**Table 4:** Continuous *Y* and logit selection model: Simulation results for *β*_1_=1 estimates

Methods	$\beta _{Y}^{sl}$	*%* *R* *b* *i* *a* *s*	*S* *E*_*cal*_	*S* *E*_*emp*_	RMSE	Cover
Before	0	0.2	0.063	0.064	0.064	95.4
deletion	1	0.0	0.064	0.066	0.066	94.2
	2	-0.2	0.063	0.061	0.061	96.2
CCA	0	0.3	0.079	0.078	0.078	95.8
	1	-18.9	0.079	0.080	0.205	33.5
	2	-30.1	0.075	0.076	0.310	2.1
HEml	0	0.3	0.105	0.108	0.108	93.6
	1	-1.3	0.117	0.131	0.131	92.7
	2	-1.5	0.098	0.111	0.112	94.3
MIHEml	0	0.3	0.107	0.110	0.110	94.0
	1	-1.2	0.121	0.133	0.134	92.6
	2	-1.3	0.105	0.113	0.114	94.7
HE2steps	0	0.3	0.107	0.105	0.105	95.4
	1	0.9	0.149	0.158	0.158	95.0
	2	0.0	0.162	0.165	0.165	95.6
MIHE2steps	0	0.3	0.110	0.106	0.106	95.5
	1	0.9	0.151	0.159	0.159	94.6
	2	0.0	0.163	0.166	0.166	94.8

*%Rbias*: % relative bias; *SE*_*cal*_: Root mean square of the estimated standard error; *SE*_*emp*_: Empirical Monte Carlo standard error; RMSE: Root mean square error; Cover: % coverage of the nominal 95% confidence interval; CCA: Complete case analysis; HEml: Heckman one-step ML estimation; MIHEml: Multiple imputation using Heckman’s one-step ML estimation; HE2steps: Heckman’s two-step estimation; MIHE2steps: Multiple imputation using Heckman’s two-step estimation

**Table 5:** Binary *Y*: Simulation results for *β*_1_=1 with *ρ*=0, representing a MAR mechanism, and *ρ*=0.3 and 0.6, representing an MNAR mechanism, in the presence of missing data on *X*_2_

		*R*_2_ depends on *X*_1_ and *X*_3_				*R*_2_ depends on *X*_1_ and *Y*			
Methods	*ρ*	*%* *R* *b* *i* *a* *s*	*S* *E*_*cal*_	RMSE	Cover	*%* *R* *b* *i* *a* *s*	*S* *E*_*cal*_	RMSE	Cover
Before deletion	0	0.7	0.109	0.113	95.0	0.8	0.109	0.111	94.9
	0.3	0.5	0.108	0.108	95.7	0.9	0.109	0.109	95.0
	0.6	0.5	0.108	0.106	95.8	1.1	0.109	0.106	95.5
CCA	0	1.0	0.158	0.159	95.3	-20.3	0.165	0.262	73.9
	0.3	-3.8	0.158	0.166	93.4	-26.9	0.164	0.313	60.9
	0.6	-8.4	0.158	0.178	90.9	-33.2	0.165	0.369	47.6
HEml	0	-1.3	0.182	0.182	94.8	-21.1	0.190	0.287	78.5
	0.3	-0.5	0.169	0.175	94.3	-21.2	0.178	0.274	76.3
	0.6	-1.1	0.158	0.158	95.0	-22.5	0.165	0.277	73.8
MIHEml	0	-2.1	0.167	0.167	95.2	-1.4	0.166	0.168	94.5
	0.3	-1.8	0.155	0.153	95.6	-1.7	0.155	0.151	95.7
	0.6	-2.5	0.146	0.140	96.6	-2.5	0.145	0.140	96.0

*%Rbias*: % relative bias; *SE*_*cal*_: Root mean square of the estimated standard error; *SE*_*emp*_: Empirical Monte Carlo standard error; RMSE: Root mean square error; Cover: % coverage of the nominal 95% confidence interval; CCA: Complete case analysis; HEml: Heckman’s one-step ML estimation; MIHEml: Multiple imputation using Heckman’s one-step ML estimation

**Table 6:** Continuous *Y*: Simulation results for *β*_1_=1 with *ρ*=0, representing a MAR mechanism, and *ρ*=0.3 and 0.6, representing an MNAR mechanism, in the presence of missing data on *X*_2_

		*R*_2_ depends on *X*_1_ and *X*_3_				*R*_2_ depends on *X*_1_ and *Y*			
Methods	*ρ*	*%* *R* *b* *i* *a* *s*	*S* *E*_*cal*_	RMSE	Cover	*%* *R* *b* *i* *a* *s*	*S* *E*_*cal*_	RMSE	Cover
Before deletion	0	0.2	0.063	0.063	94.6	-0.1	0.063	0.064	95.8
	0.3	0.1	0.064	0.065	94.8	0.0	0.063	0.064	94.7
	0.6	-0.3	0.064	0.065	94.0	0.2	0.063	0.063	95.2
CCA	0	0.1	0.095	0.092	94.8	-28.9	0.097	0.307	15.9
	0.3	-6.3	0.093	0.118	87.4	-33.8	0.094	0.351	4.7
	0.6	-13.3	0.090	0.162	69.7	-37.7	0.090	0.388	1.6
HEml	0	0.1	0.113	0.112	94.3	-27.7	0.118	0.306	34.6
	0.3	-0.3	0.110	0.121	92.7	-26.9	0.110	0.294	34.4
	0.6	-0.6	0.103	0.106	93.3	-27.5	0.099	0.293	20.9
MIHEml	0	-0.1	0.107	0.103	95.0	1.2	0.111	0.107	95.8
	0.3	-0.9	0.105	0.109	94.1	-0.1	0.108	0.107	93.7
	0.6	-2.2	0.101	0.099	94.9	-1.7	0.103	0.097	94.2
HE2steps	0	0.2	0.114	0.111	94.8	-28.1	0.117	0.306	30.8
	0.3	0.0	0.114	0.123	92.7	-27.7	0.111	0.300	29.2
	0.6	-0.3	0.113	0.114	93.9	-28.0	0.104	0.299	23.7
MIHE2steps	0	-0.1	0.107	0.101	95.5	1.1	0.110	0.107	96.1
	0.3	-0.6	0.106	0.112	93.1	0.0	0.108	0.110	94.7
	0.6	-1.5	0.105	0.105	94.8	-1.3	0.105	0.102	94.5

*%Rbias*: % relative bias; *SE*_*cal*_: Root mean square of the estimated standard error; *SE*_*emp*_: Empirical Monte Carlo standard error; RMSE: Root mean square error; Cover: % coverage of the nominal 95% confidence interval; CCA: Complete case analysis; HEml: Heckman one-step ML estimation; MIHEml: Multiple imputation using Heckman’s one-step ML estimation; HE2steps: Heckman’s two-step estimation; MIHE2steps: Multiple imputation using Heckman’s two-step estimation

The results of the simulations with data created using a logit selection model including *Y* as a covariate (i.e., a non-Heckman MNAR mechanism) are presented in Table 3 (Fig. 1) for binary outcomes and in Table 4 (Fig. 2) for continuous outcomes. *CCA* is not biased for $\beta ^{sl}_{Y}=0$ and is biased for $\beta ^{sl}_{Y} \neq 0$. The biases increase with the effect of *Y*. For MNAR binary outcomes, *HEml* and *MIHEml* are biased from 2.5 to 4.2% but are less biased than *CCA*. For continuous outcomes, *HEml*, *HE*2*steps*, *MIHEml* and *MIHE*2*steps* are slightly biased for $\beta ^{sl}_{Y}\neq 0$, and lower standard errors are obtained using *HEml* and *MIHEml*, while biases appear to be very slightly greater.

### Missing data on outcome *Y* and covariate *X*_2_

The results of the simulations that considered missing data on a binary outcome *Y* and on *X*_2_ depending on *X*_1_ and *X*_3_ are presented in Table 5 (Fig. 1). Approximately 50% of the cases were analysed with *CCA*, while 70% were analysed with *HEml* and the entire dataset with *MIHEml*. Under a MAR mechanism for the missing outcome (*ρ*=0), the biases for *CCA*, *HEml* and *MIHEml* range from 1.0 to 2.1%. The smallest standard error is obtained using *CCA*. If the missing mechanism is MNAR, then *CCA* is biased from 3.8 to 8.4%, whereas the biases of *HEml* and *MIHEml* remain less than 2.5%. *MIHEml* provides lower standard errors than *HEml* notably because *HEml* uses only approximately 70% of the observations. The results for *β*_2_ are similar (Additional file 1: Table S10).

The results of the simulations that considered missing data on binary outcome *Y* and on *X*_2_ depending on *X*_1_ and *Y* are presented in Table 5 (Fig. 1). Regardless of *ρ*, *CCA* and *HEml* are biased from 20% to more than 33%. Regardless of *ρ*, *MIHEml* is almost unbiased (relative bias of less than 2.5%). The results for *β*_2_ are similar excepted for unbiased *HEml* (Additional file 1: Table S10).

The results of the simulation studies with missing continuous outcomes *Y* and missing *X*_2_ depending on *X*_1_ and *X*_3_ are presented for *β*_1_ in Table 6 (Fig. 2). When *ρ*=0, all methods are unbiased (relative biases of less than 1%). The smallest standard error is obtained with *CCA*. When *ρ*≠0, *CCA* is biased from 6.3 to 13.3%. The other methods are almost unbiased (relative biases of less than 2.2%). The results for *β*_2_ are similar (Additional file 1: Table S11).

The results of the simulations with missing continuous outcomes *Y* and missingness of *X*_2_ depending on *X*_1_ and *Y* are presented for *β*_1_ in Table 6 (Fig. 2). Regardless of *ρ*, *CCA*, *HEml* and *HE*2*steps* are biased from 27.7% to more than 37.7%. *CCA* presents the smallest standard error. Regardless of *ρ*, *MIHEml* and *MIHE*2*steps* are unbiased (relative biases of less than 2%). The standard errors observed for *MIHEml* are smaller than those observed for *MIHE*2*steps*, while the coverage remains close to 95%. The results for *β*_2_ are similar (Additional file 1: Table S11). However, when *ρ*=0.6, *MIHEml* and *MIHE*2*steps* are slightly biased for *β*_2_ (3.4% and 4.5%, respectively).

Similar results are observed when the sample size decreased down to 200, although biases ans SEs slightly increased (Additional file 1: Tables S12, S13, S14 and S15).

## Application to illustrative examples

The impact of *treatment group* on *adherence* has been assessed using a probit model adjusted on *severity score*. *Adherence* presented 115 (21%) missing data. There were 51 and 375 non-adherent and adherent patients, respectively. The missing data mechanism of *adherence* was strongly suspected to be MNAR. The severity score was missing for 114 (21%) patients, and its missing data mechanism was suspected to be MAR. Four methods were applied: *CCA*, *HEml*, *MIHEml* and *MI*. A standard *MI* approach was added using a MICE procedure with a linear imputation model for *severity score* and a probit imputation model for *adherence*. The aim of the latter model was to assess the performance of an available misspecified but widely used approach. The missing data mechanisms assumed by each method are presented in Table 7. The *HEml* and *MIHEml* selection equations for *adherence* included *treatment group*, *severity score* and *antibiotic treatment*. The latter binary variable was chosen to fulfill the exclusion-restriction criterion. The MAR variables were imputed using linear and probit regression models for continuous and binary variables, respectively. Using *MIHEml*, the indicator of *adherence* missingness was included in the *severity score* imputation model. The MICE procedure was applied for 20 iterations, and *m*=100 datasets were generated. Finally, Rubin’s rules for small samples were applied.

**Table 7:** Estimation of the predictive value of the randomisation group and severity score

Methods (% used)	Assumed mechanisms		*Oseltamivir Placebo*		*Zanamivir Placebo*		*Severity score*	
	*Adh.*	*Sev.*	Coeff	SE	Coeff	SE	Coeff	SE
CCA (66%)	MCAR	MCAR	0.243	0.217	0.061	0.206	0.021	0.163
MI (100%)	MAR	MAR	0.380	0.205	0.055	0.183	0.035	0.163
HEml (79%)	MNAR	MCAR	0.272	0.268	0.077	0.223	0.048	0.223
MIHEml (100%)	MNAR	MAR	0.396	0.188	0.105	0.182	0.123	0.181

Adh.: Adherence; Sev.: Severity score; Coeff: Coefficient; SE: Standard error; CCA: Complete case analysis; MI: Multiple imputation using classic imputation models; HEml: Heckman’s one-step ML estimation; MIHEml: Multiple imputation using Heckman’s one-step ML estimation

The results are presented in Table 7. The reference group for *treatment* is the combination group. The *Severity score* coefficient corresponds to an increase of 20 units. *CCA* includes only 359 cases, i.e., 66% of the entire dataset. Observations with missing predictors are ignored in the *HEml* analyses, i.e., only 427 (79%) cases are retained. *MI* and *MIHEml* consider all observations. As expected, *MI* and *MIHEml* have lower standard errors than those of *CCA* and *HEml*. The coefficients estimated for *Oseltamivir-Placebo* with *MI* and *MIHEml* are similar and higher than those obtained with *CCA* or *HEml*. The effect of *Oseltamivir-Placebo* reached significance with *MIHEml*, thus enabling the assessment of the impact of *Oseltamivir-Placebo* on *adherence*. The estimated coefficients of *Zanamivir-Placebo* and *severity score* are similar for *CCA* and *MI*, slightly higher for *HEml* and higher for *MIHEml*. Not surprisingly, the proportion of imputed values corresponding to the non-adherent outcome are 13% and 47% for *MI* and *MIHEml*, respectively, indicating that missing values on self-reported adherence are more likely to correspond to non-adherent patients.

We also challenged the MAR assumption concerning the missing mechanism associated with the *severity score*. Thus, we performed a new MICE procedure encoding two Heckman’s imputation models for *adherence* and *severity score*. It involves defining selection and outcome models for *severity score*. The results for the effects were similar: 0.376 (0.186) and 0.096 (0.179) for *Oseltamivir-Placebo* and *Zanamivir-Placebo*, respectively. These results suggest a weak impact of the MNAR mechanism for *severity score*.

## Discussion

The first aim of this work was to propose a unique approach to address binary outcomes according to an MNAR mechanism and missing predictors with a MAR mechanism. According to our simulation results, for MNAR outcomes, only *MIHEml* and *HEml* were unbiased. Our simulation studies were generated using a real Heckman’s model. Thus, we generated MNAR outcomes using a logistic selection model, directly including *Y* as a predictor, i.e. an MNAR mechanism that is non-compatible with Heckman’s model. Although our results remain biased, the use of *MIHEml* reduced the biases compared to *CCA*. Because it is not possible to confirm the validity of Heckman’s model from the observed data alone [17, 33], the developed approach appears to at least reduce the biases under an MNAR mechanism if the Heckman’s hypotheses do not hold.

To thoroughly evaluate our approach in a MICE procedure, we simulated missing data on predictors following two scenarios: one where the MAR mechanism for *X*_2_ depended on the fully observed *X*_1_ and *X*_3_, and one where the mechanism depended on *X*_1_ and *Y*. For these two scenarios, Heckman’s model (*HEml*) used only cases with complete predictors to estimate the model parameters, i.e, did not use all available information. This loss of information produced larger standard errors, particularly for *β*_1_ and only slightly for *β*_2_. This result is not surprising because the information lost, resulting from ignoring patients with missing *X*_2_, primarily affected *X*_1_. In terms of bias, the first scenario presented similar results to those obtained without missing *X*_2_ data. In the second scenario, where the missing mechanism for *X*_2_ also depended on *Y*, *MIHEml* out-performed all the other methods. The second aim was to validate the proposition of Galimard et al. [18] using a one-step ML estimator for continuous outcome. Our simulations showed that *MIHEml* performs slightly better than *MIHE*2*steps* in terms of standard errors for the missing MNAR outcomes.

Although our method performs well in the presence of a MAR mechanism, i.e., when *ρ*=0, it is preferable to determine whether the missing data mechanism is most likely to be MNAR or MAR to avoid modelling a selection equation. Indeed, the standard errors are greater than those of the standard approaches for *ρ*=0. Unfortunately, it is not possible to distinguish between MAR and MNAR from the observed data alone [17, 33]. Hence, sensitivity analyses are often performed to evaluate departures from MAR. Some authors have proposed a pattern mixture model using *δ* adjustment, i.e., systematically adding a certain increment *δ* to the linear predictors of the imputed values. Despite its simplicity, van Buuren considered this method to be a powerful approach for evaluating the MAR mechanism by varying *δ* [2, 8, 17]. This method identifies two patterns: one for the observed data and one for the unobserved data. Missing values are imputed conditionally on the observed data with an additional shift parameter *δ*, which is the magnitude of departure from MAR. Then, the model for the observed data is different from the model for the missing data. Similarly, *MIHEml* can be viewed as a method that applies a shift term or a correction term for the selection bias in the imputation model specific to each observation *i*. Precisely, as $E(Y_{i}|R_{yi}=0)=X_{i}\beta +\rho \sigma _{\varepsilon }\left (-\phi \left (X^{s}_{i}\beta ^{s}\right)\right)/\Phi \left (-X^{s}_{i}\beta ^{s}\right)$, *MIHEml* uses a selection correction term that can be considered as an individual *δ*_*i*_ for each patient (adjusted on the parameters of the selection equation). In this sense, we obtained a more precise *δ*-adjustment approach.

The construction of the selection model follows strict rules [14, 23]. In our experience, respect of the exclusion-restriction criterion should be strict. Indeed, Heckman’s model can inflate standard errors due to the collinearity between the regressors and *IMR*, and this problem is exacerbated when the exclusion-restriction criterion does not hold [34]. Moreover, MICE (or full conditional specification) follows certain rules. Each variable with missing data requires a specific conditional imputation model that is generally defined by a link function and a linear predictor with its set of predictors. Theoretically, imputation models should be derived from the global joint distribution of the variables, including the outcome [2, 35], and misspecification may result in biased parameter estimates [36]. Despite recent work in simple cases, the theoretical properties of MICE are not fully understood [25, 26, 28, 37]. Nevertheless, it performs well in practice, particularly when the conditional imputation models are well accommodated to the substantive model. The efficiency of the MICE approach is generally validated by simulation studies, and the results appear robust even when the compatibility between the full conditional distribution and the global joint distribution is not proven [2]. Although simulation is never sufficiently complete, these simulations suggest that our approach of multiple imputation using Heckman’s model and its use in a MICE process are valid and could be useful when the MNAR mechanism on the outcome is compatible with Heckman’s model. To avoid the bivariate normality assumption of Heckman’s model, Marchenko and Genton [38] proposed a Heckman’s model with a bivariate Student distribution for error terms. Ogundimu and Collins [39] developed an imputation model using this selection-t model. Unfortunately, their imputation model is only available for continuous outcomes. We compare the proposition in the current paper for continuous outcome to the propositions of Ogundimu and Collins [39] and Galimard et al. [18] in Additional file 2. Not surprisingly, the results were similar. Indeed, *t*-distributions are very close to a normal distribution for high degrees of freedom. In this paper, we focused on frequentist sample selection approaches within a MICE procedure. Nevertheless, Bayesian posterior distribution of sample selection models can be obtained using Gibbs sampling and data augmentation [40, 41]. Such a fully Bayesian framework could improve the imputation when based on small samples; this could be evaluated in further research.

Finally, our simulation study does not explore MNAR mechanisms on covariates and outcomes. Such a situation requires specifying a Heckman’s imputation model for each MNAR variable (i.e. selection and outcome models). Nevertheless, we used this type of approach in our example analysis to evaluate the departure from MAR for the missing predictors.

## Conclusion

In the presence of MAR predictors, we proposed a simple approach to address MNAR binary or continuous missing outcomes under a Heckman assumption in a MICE procedure. This approach could be either directly used to handle such a framework (MNAR outcomes and MAR predictors) or to challenge the robustness of a suspected MAR mechanism for missing outcomes, such as in a sensitivity analysis. Finally, a R package, named “miceMNAR”, dedicated to the proposed approaches has been implemented and is available on the CRAN (https://CRAN.R-project.org/package=miceMNAR).

## Additional files

Additional file 1 Additional tables. (PDF 108 kb)

Additional file 2 Comparison to Ogundimu and Collins. (PDF 129 kb)

Additional file 3 BIVIR study group. (PDF 78 kb)

Additional file 4 R code to impute binary outcome. (R 1 kb)

Additional file 5 R code to impute continuous outcome. (R 1 kb)

## Abbreviations

CCA: Complete case analysis
Cover: Coverage of nominal 95% confidence intervals
HEml: Heckman’s one-step ML estimation
HE2steps: Heckman’s two-step estimation
IMR: Inverse Mills ratio
MAR: Missing at random
MCAR: Missing completely at random
MNAR: Missing not at random
MI: Multiple imputation
MICE: Multiple imputation by chained equations
MIHEml: Multiple imputation using Heckman’s one-step ML estimation
MIHE2steps: Multiple imputation using Heckman’s two-step estimation
Rbias: Relative bias
RMSE: Root mean square error
*S**E*_*cal*_: Root mean square of estimated standard errors
*S**E*_*emp*_: Empirical Monte Carlo standard errors
