# Supplementary material for: Heckman imputation models for binary or continuous MNAR outcomes and MAR predictors
Source: BMC Med Res Methodol. 2018 Aug 31;18:90. doi: 10.1186/s12874-018-0547-1 (PMC6119269; doi:10.1186/s12874-018-0547-1)
Supplement: Supplementary file 1 — Additional tables. (PDF 108 kb) [file 12874_2018_547_MOESM1_ESM.pdf]

Heckman imputation models for binary or continuous MNAR outcomes and MAR predictors

J.-E. Galimard, S. Chevret, E. Curis and M. Resche-Rigon

Additional file 1: Additional tables

This additional file contains 8 supplementary tables :

Table S8. Binary Y: Simulation results for $\beta_2 = 1$ with $\rho = 0$, representing a MAR mechanism, and $\rho = 0.3$ and 0.6, representing an MNAR mechanism.

Table S9. Continuous Y: Simulation results for $\beta_2 = 1$ with $\rho = 0$, representing a MAR mechanism, and $\rho = 0.3$ and 0.6, representing an MNAR mechanism.

Table S10. Binary Y: Simulation results for $\beta_2 = 1$ with $\rho = 0$, representing a MAR mechanism, and $\rho = 0.3$ and 0.6, representing an MNAR mechanism, in the presence of missing data on X_2.

Table S11. Continuous Y: Simulation results for $\beta_2 = 1$ with $\rho = 0$, representing a MAR mechanism, and $\rho = 0.3$ and 0.6, representing an MNAR mechanism, in the presence of MAR missing data on X_2.

Table S12. Binary Y: Simulation results for n=200, $\beta_1 = 1$ with $\rho = 0$, representing a MAR mechanism, and $\rho = 0.3$ and 0.6, representing an MNAR mechanism.

Table S13. Continuous Y: Simulation results for n=200, $\beta_1 = 1$ with $\rho = 0$, representing a MAR mechanism, and $\rho = 0.3$ and 0.6, representing an MNAR mechanism.

Table S14. Binary Y: Simulation results for n=200, $\beta_1 = 1$ with $\rho = 0$, representing a MAR mechanism, and $\rho = 0.3$ and 0.6, representing an MNAR mechanism, in the presence of missing data on X_2.

Table S15. Continuous Y: Simulation results for n=200, $\beta_1 = 1$ with $\rho = 0$, representing a MAR mechanism, and $\rho = 0.3$ and 0.6, representing an MNAR mechanism, in the presence of missing data on X_2.

Table S8: Binary Y: Simulation results for $\beta_2 = 1$ with $\rho = 0$, representing a MAR mechanism, and $\rho = 0.3$ and 0.6, representing an MNAR mechanism.

Methods	ρ	$\%Rbias$	SE_{cal}	SE_{emp}	RMSE	Cover
Before deletion	0	1.1	0.109	0.109	0.109	95.1
	0.3	1.2	0.109	0.110	0.111	95.7
	0.6	0.9	0.109	0.112	0.112	94.8
CCA	0	1.2	0.133	0.134	0.134	94.5
	0.3	7.2	0.135	0.135	0.153	93.7
	0.6	16.7	0.140	0.150	0.224	80.7
HEml	0	-0.1	0.140	0.140	0.140	94.8
	0.3	0.4	0.149	0.145	0.145	95.5
	0.6	0.8	0.156	0.165	0.166	93.5
MIHEml	0	-1.0	0.139	0.138	0.138	94.1
	0.3	-0.2	0.148	0.142	0.142	95.5
	0.6	0.9	0.157	0.162	0.162	93.9

$\%Rbias$: % relative bias; SE_{cal}: Root mean square of the estimated standard error; SE_{emp}: Empirical Monte Carlo standard error; RMSE: Root mean square error; Cover: % coverage of the nominal 95% confidence interval; CCA: Complete case analysis; HEml: Heckman's one-step ML estimation; MIHEml: Multiple imputation using Heckman's one-step ML estimation.

Table S9: Continuous Y: Simulation results for $\beta_2 = 1$ with $\rho = 0$, representing a MAR mechanism, and $\rho = 0.3$ and 0.6, representing an MNAR mechanism.

Methods	ρ	$\%Rbias$	SE_{cal}	SE_{emp}	RMSE	Cover
Before deletion	0	-0.1	0.064	0.065	0.065	93.9
	0.3	0.0	0.063	0.063	0.063	95.2
	0.6	-0.1	0.064	0.066	0.066	93.5
CCA	0	-0.3	0.079	0.079	0.079	94.7
	0.3	4.4	0.078	0.077	0.089	91.1
	0.6	8.9	0.075	0.077	0.117	77.8
HEml	0	-0.4	0.085	0.086	0.086	94.4
	0.3	0.1	0.083	0.085	0.085	94.9
	0.6	0.2	0.080	0.082	0.082	93.6
MIHEml	0	-0.4	0.086	0.087	0.087	94.6
	0.3	0.0	0.086	0.086	0.086	95.2
	0.6	0.1	0.084	0.082	0.082	94.6
HE2steps	0	-0.4	0.085	0.085	0.085	95.1
	0.3	0.1	0.084	0.086	0.086	95.3
	0.6	0.1	0.083	0.084	0.084	94.6
MIHE2steps	0	-0.4	0.086	0.085	0.085	95.5
	0.3	0.1	0.085	0.086	0.086	95.0
	0.6	0.1	0.084	0.084	0.084	95.0

$\%Rbias$: % relative bias; SE_{cal}: Root mean square of the estimated standard error; SE_{emp}: Empirical Monte Carlo standard error; RMSE: Root mean square error; Cover: % coverage of the nominal 95% confidence interval; CCA: Complete case analysis; HEml: Heckman one-step ML estimation; MIHEml: Multiple imputation using Heckman's one-step ML estimation; HE2steps: Heckman's two-step estimation; MIHE2steps: Multiple imputation using Heckman's two-step estimation.

Table S10: Binary Y: Simulation results for $\beta_2 = 1$ with $\rho = 0$, representing a MAR mechanism, and $\rho = 0.3$ and 0.6, representing an MNAR mechanism, in the presence of missing data on X_2.

		R_2 depends on X_1 and X_3				R_2 depends on X_1 and Y			
Methods	ρ	$\%Rbias$	SE_{cal}	RMSE	Cover	$\%Rbias$	SE_{cal}	RMSE	Cover
Before deletion	0	1.0	0.109	0.110	95.6	0.4	0.109	0.113	93.9
	0.3	0.9	0.109	0.112	94.6	1.3	0.109	0.110	95.5
	0.6	1.2	0.109	0.109	95.6	0.7	0.109	0.109	94.2
CCA	0	1.6	0.149	0.150	95.1	0.9	0.153	0.156	95.1
	0.3	7.5	0.153	0.177	92.9	7.3	0.158	0.180	93.3
	0.6	16.0	0.158	0.228	84.8	15.0	0.165	0.223	88.1
HEml	0	-0.9	0.160	0.161	94.4	-1.1	0.163	0.161	94.8
	0.3	0.4	0.170	0.175	94.3	0.1	0.174	0.176	94.7
	0.6	1.3	0.177	0.181	94.4	-0.2	0.181	0.185	94.2
MIHEml	0	-1.6	0.155	0.151	94.9	-1.8	0.161	0.159	95.2
	0.3	-0.6	0.162	0.162	95.7	-0.6	0.169	0.167	95.7
	0.6	1.5	0.171	0.164	95.8	0.1	0.177	0.169	95.8

$\%Rbias$: % relative bias; SE_{cal}: Root mean square of the estimated standard error; SE_{emp}: Empirical Monte Carlo standard error; RMSE: Root mean square error; Cover: % coverage of the nominal 95% confidence interval; CCA: Complete case analysis; HEml: Heckman's one-step ML estimation; MIHEml: Multiple imputation using Heckman's one-step ML estimation.

Table S11: Continuous Y: Simulation results for $\beta_2 = 1$ with $\rho = 0$, representing a MAR mechanism, and $\rho = 0.3$ and 0.6, representing an MNAR mechanism, in the presence of MAR missing data on X_2.

		R_2 depends on X_1 and X_3				R_2 depends on X_1 and Y			
Methods	ρ	%*Rbias*	SE_{cal}	RMSE	Cover	%*Rbias*	SE_{cal}	RMSE	Cover
Before	0	0.2	0.063	0.063	95.2	0.0	0.063	0.062	95.1
deletion	0.3	-0.2	0.063	0.062	95.3	-0.3	0.063	0.062	95.0
	0.6	0.0	0.064	0.062	95.3	-0.1	0.063	0.062	95.5
CCA	0	0.4	0.087	0.085	95.9	-14.9	0.086	0.171	58.6
	0.3	3.6	0.086	0.092	93.5	-10.8	0.084	0.137	75.0
	0.6	8.1	0.083	0.114	84.4	-6.4	0.081	0.104	87.4
HEml	0	0.4	0.095	0.096	94.6	-15.6	0.094	0.181	62.1
	0.3	0.0	0.093	0.091	95.5	-15.2	0.093	0.179	61.3
	0.6	0.5	0.090	0.088	95.3	-14.9	0.089	0.175	60.1
MIHEml	0	0.4	0.092	0.089	94.6	-1.3	0.100	0.095	96.9
	0.3	-0.3	0.090	0.087	95.5	-2.0	0.098	0.094	95.0
	0.6	-0.2	0.089	0.085	95.3	-3.4	0.094	0.095	95.3
HE2steps	0	0.4	0.096	0.096	94.4	-15.3	0.093	0.177	62.1
	0.3	-0.2	0.095	0.092	95.8	-14.6	0.092	0.173	65.1
	0.6	0.4	0.094	0.090	95.4	-14.2	0.092	0.169	66.9
MIHE2steps	0	0.5	0.091	0.089	94.7	-1.1	0.099	0.095	96.2
	0.3	-0.3	0.091	0.087	94.5	-2.2	0.099	0.097	95.1
	0.6	-0.2	0.091	0.086	96.0	-4.5	0.098	0.105	93.6

%*Rbias*: % relative bias; SE_{cal}: Root mean square of the estimated standard error; SE_{emp}: Empirical Monte Carlo standard error; RMSE: Root mean square error; Cover: % coverage of the nominal 95% confidence interval; CCA: Complete case analysis; HEml: Heckman one-step ML estimation; MIHEml: Multiple imputation using Heckman's one-step ML estimation; HE2steps: Heckman's two-step estimation; MIHE2steps: Multiple imputation using Heckman's two-step estimation.

Table S12: Binary Y: Simulation results for n=200, $\beta_1 = 1$ with $\rho = 0$, representing a MAR mechanism, and $\rho = 0.3$ and 0.6, representing an MNAR mechanism.

Methods	ρ	$\%Rbias$	SE_{cal}	SE_{emp}	RMSE	Cover
Before deletion	0	1.8	0.174	0.182	0.183	93.8
	0.3	3.8	0.176	0.182	0.186	95.5
	0.6	2.3	0.175	0.183	0.184	94.8
CCA	0	3.5	0.222	0.237	0.239	94.5
	0.3	-3.0	0.220	0.228	0.230	94.1
	0.6	-9.8	0.219	0.237	0.256	89.6
HEml	0	0.0	0.253	0.271	0.271	91.9
	0.3	0.7	0.239	0.239	0.238	94.4
	0.6	0.7	0.221	0.234	0.234	94.8
MIHEml	0	-1.1	0.248	0.260	0.260	93.9
	0.3	-0.9	0.237	0.234	0.234	95.2
	0.6	-1.3	0.223	0.234	0.234	95.1

$\%Rbias$: % relative bias; SE_{cal}: Root mean square of the estimated standard error; SE_{emp}: Empirical Monte Carlo standard error; RMSE: Root mean square error; Cover: % coverage of the nominal 95% confidence interval; CCA: Complete case analysis; HEml: Heckman's one-step ML estimation; MIHEml: Multiple imputation using Heckman's one-step ML estimation.

Table S13: Continuous Y: Simulation results for n=200, $\beta_1 = 1$ with $\rho = 0$, representing a MAR mechanism, and $\rho = 0.3$ and 0.6, representing an MNAR mechanism.

Methods	ρ	$\%Rbias$	SE_{cal}	SE_{emp}	RMSE	Cover
Before deletion	0	-0.7	0.101	0.102	0.102	95.2
	0.3	0.7	0.101	0.100	0.101	94.6
	0.6	-0.2	0.101	0.096	0.096	96.2
CCA	0	-0.8	0.132	0.132	0.132	95.0
	0.3	-8.1	0.131	0.124	0.148	90.9
	0.6	-17.6	0.125	0.123	0.215	70.9
HE2steps	0	-0.6	0.166	0.170	0.170	94.1
	0.3	0.9	0.165	0.158	0.158	95.7
	0.6	-0.4	0.161	0.159	0.159	95.4
MIHE2steps	0	-0.6	0.172	0.172	0.172	94.8
	0.3	0.7	0.171	0.159	0.159	96.5
	0.6	-0.3	0.168	0.159	0.159	95.4
HEml	0	-0.6	0.163	0.173	0.173	92.6
	0.3	0.7	0.159	0.156	0.156	94.3
	0.6	-0.7	0.147	0.152	0.152	93.5
MIHEml	0	-0.5	0.168	0.176	0.176	93.8
	0.3	0.8	0.166	0.158	0.159	95.1
	0.6	-0.5	0.157	0.154	0.154	94.2

$\%Rbias$: % relative bias; SE_{cal}: Root mean square of the estimated standard error; SE_{emp}: Empirical Monte Carlo standard error; RMSE: Root mean square error; Cover: % coverage of the nominal 95% confidence interval; CCA: Complete case analysis; HEml: Heckman one-step ML estimation; MIHEml: Multiple imputation using Heckman's one-step ML estimation; HE2steps: Heckman's two-step estimation; MIHE2steps: Multiple imputation using Heckman's two-step estimation.

Table S14: Binary Y: Simulation results for n=200, $\beta_1 = 1$ with $\rho = 0$, representing a MAR mechanism, and $\rho = 0.3$ and 0.6, representing an MNAR mechanism, in the presence of missing data on X_2.

		R_2 depends on X_1 and X_3				R_2 depends on X_1 and Y			
Methods	ρ	$\%Rbias$	SE_{cal}	RMSE	Cover	$\%Rbias$	SE_{cal}	RMSE	Cover
Before	0	3.1	0.175	0.183	95.1	2.8	0.175	0.185	94.8
deletion	0.3	2.7	0.175	0.185	95.1	3.0	0.175	0.179	95.3
	0.6	2.4	0.175	0.183	94.4	2.5	0.175	0.188	93.9
CCA	0	5.2	0.259	0.266	96.1	-17.5	0.269	0.332	87.3
	0.3	-2.4	0.256	0.263	94.5	-25.8	0.267	0.382	79.4
	0.6	-4.0	0.259	0.281	94.6	-31.0	0.268	0.415	73.8
HEml	0	0.1	0.279	0.281	93.4	-20.1	0.293	0.368	85.4
	0.3	-2.2	0.267	0.274	93.3	-21.0	0.283	0.358	87.4
	0.6	0.7	0.258	0.271	94.3	-21.7	0.268	0.349	84.6
MIHEml	0	-1.0	0.260	0.249	95.1	-1.9	0.260	0.251	95.2
	0.3	-3.7	0.248	0.238	96.1	-2.5	0.248	0.248	94.1
	0.6	-2.2	0.238	0.232	96.4	-3.5	0.237	0.241	95.0

$\%Rbias$: % relative bias; SE_{cal}: Root mean square of the estimated standard error; SE_{emp}: Empirical Monte Carlo standard error; RMSE: Root mean square error; Cover: % coverage of the nominal 95% confidence interval; CCA: Complete case analysis; HEml: Heckman's one-step ML estimation; MIHEml: Multiple imputation using Heckman's one-step ML estimation.

Table S15: Continuous Y: Simulation results for n=200, $\beta_1 = 1$ with $\rho = 0$, representing a MAR mechanism, and $\rho = 0.3$ and 0.6, representing an MNAR mechanism, in the presence of missing data on X_2.

		R_2 depends on X_1 and X_3				R_2 depends on X_1 and Y			
Methods	ρ	%*Rbias*	SE_{cal}	RMSE	Cover	%*Rbias*	SE_{cal}	RMSE	Cover
Before	0	-0.1	0.1	0.1	93.4	0.3	0.101	0.099	95.0
deletion	0.3	0.1	0.101	0.098	95.3	0.0	0.101	0.103	94.5
	0.6	-0.2	0.101	0.099	95.8	-0.1	0.101	0.100	95.0
CCA	0	-0.2	0.15	0.146	95.9	-29.2	0.156	0.333	53.3
	0.3	-6.6	0.149	0.16	93.1	-34.4	0.150	0.376	36.9
	0.6	-12.5	0.145	0.194	87.5	-37.9	0.143	0.406	25.2
HE2steps	0	-0.5	0.184	0.185	96.1	-28.2	0.189	0.347	65.0
	0.3	0.2	0.185	0.184	95.8	-28.4	0.177	0.336	62.8
	0.6	-0.2	0.182	0.185	94.8	-27.3	0.168	0.326	61.1
MIHE2steps	0	-0.9	0.174	0.166	96.5	1.0	0.181	0.175	96.0
	0.3	-0.6	0.174	0.164	95.8	-0.5	0.177	0.170	96.0
	0.6	-1.3	0.172	0.168	95.5	-0.9	0.173	0.171	95.0
HEml	0	-0.5	0.173	0.185	93.9	-28.0	0.183	0.350	62.4
	0.3	-0.5	0.172	0.182	94	-28.1	0.171	0.336	61.4
	0.6	-1	0.164	0.178	93.1	-27.3	0.156	0.322	58.1
MIHEml	0	-0.6	0.172	0.165	95.8	1.5	0.179	0.173	95.4
	0.3	-1.4	0.173	0.161	95.9	-0.5	0.176	0.166	95.6
	0.6	-2.3	0.167	0.157	95.6	-1.9	0.166	0.159	96.4

%*Rbias*: % relative bias; SE_{cal}: Root mean square of the estimated standard error; SE_{emp}: Empirical Monte Carlo standard error; RMSE: Root mean square error; Cover: % coverage of the nominal 95% confidence interval; CCA: Complete case analysis; HEml: Heckman one-step ML estimation; MIHEml: Multiple imputation using Heckman's one-step ML estimation; HE2steps: Heckman's two-step estimation; MIHE2steps: Multiple imputation using Heckman's two-step estimation.
