# Supplementary material for: Heckman imputation models for binary or continuous MNAR outcomes and MAR predictors
Source: BMC Med Res Methodol. 2018 Aug 31;18:90. doi: 10.1186/s12874-018-0547-1 (PMC6119269; doi:10.1186/s12874-018-0547-1)
Supplement: Supplementary file 2 — Comparison to Ogundimu and Collins. (PDF 129 kb) [file 12874_2018_547_MOESM2_ESM.pdf]

Heckman imputation models for binary or continuous MNAR outcomes and MAR predictors

J.-E. Galimard, S. Chevret, E. Curis and M. Resche-Rigon

Additional file 2: Comparison to Ogundimu and Collins

Ogundimu and Collins (2017) developed an imputation model using the selection-t model (Heckman's model with bivariate Student distribution for error terms). Marchenko and Genton (2012) proposed the previous distribution as a robust alternative to the bivariate normal distribution assumption of Heckman's model. The use of the bivariate distribution modifies the likelihood function and conditional moment. Therefore, such a distribution requires estimating a supplementary parameter: the degree of freedom. Using this distribution, Ogundimu and Collins (2017) imputed missing data from the conditional expectation of missing data as Galimard et al. (2016) but using a type of modified IMR by degree of freedom, PDF and CDF of univariate Student distribution. Unfortunately, this imputation model is only available for continuous outcomes.

We compared the proposition of the current paper to the propositions of Ogundimu and Collins (2017) and Galimard et al. (2016) using the same simulation plan performed in the current paper. Moreover, additional simulations scenarios were added:

- Generation of errors term from a bivariate Student distribution according to the following model and $\nu = 5$ degrees of freedom:

$$\begin{array}{l} R'_{yi} = X_i^s \beta^s + \varepsilon_i^s \\ Y_i = X_i \beta + \varepsilon_i \end{array}, \quad \text{with} \quad \begin{pmatrix} \varepsilon^s \\ \varepsilon \end{pmatrix} \sim T\left[\begin{pmatrix} 0 \\ 0 \end{pmatrix}, \begin{pmatrix} 1 & \rho\sigma_\varepsilon \\ \rho\sigma_\varepsilon & \sigma_\varepsilon^2 \end{pmatrix}, \nu \right] \tag{1}$$

- Absence of exclusion-restriction with $\beta_3^s = 0$.

The analysis methods were same as in the current paper for continuous case and adding the following:

- Multiple imputation using bivariate Student estimation ($MIHeckST$), as proposed and described by Ogundimu and Collins (2017) and only for continuous outcomes.

The results are presented in Tables 16, 17, 18, 19 and 20.

Table 16: Continuous Y: Simulation results for $\beta_1 = 1$ with $\rho = 0$, representing a MAR mechanism, and $\rho = 0.3$ and 0.6, representing an MNAR mechanism.

Methods	ρ	%*Rbias*	SE_{cal}	SE_{emp}	RMSE	Cover
MIHE2steps	0	0.0	0.105	0.103	0.103	95.2
	0.3	-0.4	0.104	0.104	0.104	94.0
	0.6	-0.2	0.103	0.100	0.099	95.2
MIHEml	0	0.0	0.105	0.103	0.103	94.7
	0.3	-0.3	0.103	0.102	0.102	95.3
	0.6	-0.3	0.096	0.094	0.094	94.8
MIHeckST	0	0.0	0.112	0.105	0.105	96.4
	0.3	0.0	0.110	0.103	0.103	96.1
	0.6	0.3	0.104	0.093	0.093	96.5

%*Rbias*: % relative bias; SE_{cal}: Root mean square of the estimated standard error; SE_{emp}: Empirical Monte Carlo standard error; RMSE: Root mean square error; Cover: % coverage of the nominal 95% confidence interval; MIHE2steps: Multiple imputation using Heckman's two-step estimation; MIHEml: Multiple imputation using Heckman's one-step ML estimation; MIHeckST: Multiple imputation using bivariate Student estimation

Table 17: Continuous Y: Simulation results using Heckman's model with bivariate Student distribution, for $\beta_1 = 1$ with $\rho = 0$, representing a MAR mechanism, and $\rho = 0.3$ and 0.6, representing a MNAR mechanism.

Methods	ρ	%*Rbias*	SE_{cal}	SE_{emp}	RMSE	Cover
MIHE2steps	0	0.2	0.131	0.130	0.130	94.9
	0.3	0.8	0.135	0.138	0.138	94.4
	0.6	1.4	0.133	0.129	0.130	96.2
MIHEml	0	0.2	0.137	0.132	0.131	95.2
	0.3	1.2	0.142	0.140	0.141	94.9
	0.6	4.5	0.136	0.129	0.136	95.4
MIHeckST	0	0.3	0.123	0.116	0.116	95.5
	0.3	0.4	0.125	0.118	0.118	95.5
	0.6	0.9	0.120	0.107	0.107	97.3

%*Rbias*: % relative bias; SE_{cal}: Root mean square of the estimated standard error; SE_{emp}: Empirical Monte Carlo standard error; RMSE: Root mean square error; Cover: % coverage of the nominal 95% confidence interval; MIHE2steps: Multiple imputation using Heckman's two-step estimation; MIHEml: Multiple imputation using Heckman's one-step ML estimation; MIHeckST: Multiple imputation using bivariate Student estimation

Table 18: Continuous Y: Simulation results using Heckman's model without exclusion-restriction, for $\beta_1 = 1$ with $\rho = 0$, representing a MAR mechanism, and $\rho = 0.3$ and 0.6, representing an MNAR mechanism.

Methods	ρ	$\%Rbias$	SE_{cal}	SE_{emp}	RMSE	Cover
MIHE2steps	0	-1.4	0.248	0.242	0.242	95.6
	0.3	-1.9	0.244	0.236	0.237	96.4
	0.6	-2.3	0.234	0.226	0.227	95.4
MIHEml	0	-1.8	0.164	0.187	0.187	85.8
	0.3	-5.6	0.153	0.192	0.200	83.5
	0.6	-6.4	0.131	0.187	0.197	84.8
MIHeckST	0	-1.4	0.174	0.199	0.199	85.2
	0.3	-5.3	0.163	0.200	0.207	82.9
	0.6	-5.0	0.138	0.185	0.192	88.2

$\%Rbias$: % relative bias; SE_{cal}: Root mean square of the estimated standard error; SE_{emp}: Empirical Monte Carlo standard error; RMSE: Root mean square error; Cover: % coverage of the nominal 95% confidence interval; MIHE2steps: Multiple imputation using Heckman's two-step estimation; MIHEml: Multiple imputation using Heckman's one-step ML estimation; MIHeckST: Multiple imputation using bivariate Student estimation

Table 19: Continuous Y and logit selection model: Simulation results for $\beta_1 = 1$ estimates.

Methods	β_Y^{sl}	$\%Rbias$	SE_{cal}	SE_{emp}	RMSE	Cover
MIHE2steps	0	0.3	0.110	0.106	0.106	95.5
	1	0.9	0.151	0.159	0.159	94.6
	2	0.0	0.163	0.166	0.166	94.8
MIHEml	0	0.3	0.107	0.110	0.110	94.0
	1	-1.2	0.121	0.133	0.134	92.6
	2	-1.3	0.105	0.113	0.114	94.7
MIHeckST	0	0.2	0.114	0.110	0.110	96.0
	1	-0.7	0.128	0.133	0.134	94.7
	2	-0.1	0.111	0.103	0.103	96.9

$\%Rbias$: % relative bias; SE_{cal}: Root mean square of the estimated standard error; SE_{emp}: Empirical Monte Carlo standard error; RMSE: Root mean square error; Cover: % coverage of the nominal 95% confidence interval; MIHE2steps: Multiple imputation using Heckman's two-step estimation; MIHEml: Multiple imputation using Heckman's one-step ML estimation; MIHeckST: Multiple imputation using bivariate Student estimation

Table 20: Continuous Y: Simulation results for $\beta_1 = 1$ with $\rho = 0$, representing a MAR mechanism, and $\rho = 0.3$ and 0.6, representing an MNAR mechanism, in the presence of missing data on X_2.

		R_2 depends on X_1 and X_3				R_2 depends on X_1 and Y			
Methods	ρ	$\%Rbias$	SE_{cal}	RMSE	Cover	$\%Rbias$	SE_{cal}	RMSE	Cover
MIHE2steps	0	-0.1	0.107	0.101	95.5	1.1	0.110	0.107	96.1
	0.3	-0.6	0.106	0.112	93.1	0.0	0.108	0.110	94.7
	0.6	-1.5	0.105	0.105	94.8	-1.3	0.105	0.102	94.5
MIHEml	0	-0.1	0.107	0.103	95.0	1.2	0.111	0.107	95.8
	0.3	-0.9	0.105	0.109	94.1	-0.1	0.108	0.107	93.7
	0.6	-2.2	0.101	0.099	94.9	-1.7	0.103	0.097	94.2
MIHeckST	0	-0.4	0.114	0.106	95.5	1.6	0.116	0.108	96.3
	0.3	-0.8	0.113	0.109	96.0	0.6	0.114	0.108	96.2
	0.6	-1.8	0.108	0.100	96.4	-0.9	0.108	0.094	97.2

$\%Rbias$: % relative bias; SE_{cal}: Root mean square of the estimated standard error; SE_{emp}: Empirical Monte Carlo standard error; RMSE: Root mean square error; Cover: % coverage of the nominal 95% confidence interval; MIHE2steps: Multiple imputation using Heckman's two-step estimation; MIHEml: Multiple imputation using Heckman's one-step ML estimation; MIHeckST: Multiple imputation using bivariate Student estimation

Bibliography

Galimard, J.-E., Chevret, S., Protopopescu, C., and Resche-Rigon, M. (2016). A multiple imputation approach for MNAR mechanisms compatible with Heckman's model. *Stat Med*, 35(17):2907–2920.

Marchenko, Y. V. and Genton, M. G. (2012). A heckman selection-t model. *J Am Stat Assoc*, 107(497):304–317.

Ogundimu, E. O. and Collins, G. S. (2017). A robust imputation method for missing responses and covariates in sample selection models. *Stat Meth Med Res*, 0(0).
