# Supplementary material for: Heckman imputation models for binary or continuous MNAR outcomes and MAR predictors
Source: BMC Med Res Methodol. 2018 Aug 31;18:90. doi: 10.1186/s12874-018-0547-1 (PMC6119269; doi:10.1186/s12874-018-0547-1)
Supplement: Supplementary file 3 — BIVIR study group. (PDF 78 kb) [file 12874_2018_547_MOESM3_ESM.pdf]

Heckman imputation models for binary or continuous MNAR outcomes and MAR predictors

J.-E. Galimard, S. Chevret, E. Curis and M. Resche-Rigon

Additional file 3: BIVIR study group

Scientific Committee: *Steering Committee:* Leport C (principal investigator), Andreoletti L, Blanchon T, Carrat F, Duval X, Guimfack A, Lina L, Loubière S, Mentré F, Mosnier A, Tibi A, Tubach F, van der Werf S. *Clinical Study manager:* Charlois - Ou C. *Other members:* Bricaire F, Cohen JM, Flahault A, Moatti JP, Vogel JY. *Invited members:* Eid Z (GSK), Peurichard C (GSK), Pecking M (Roche), Dantin S (Roche), Gysembergh-Houal A (AP-HP).

Independent data-monitoring committee: Chêne G, Hannoun C, Vittecoq D.

Monitoring and statistical analysis: Boucherit S, Dornic Q, Quintin C, Vincent C., and Atlanstat, Studypharm clinical research organisations.

Clinical investigators: Alea JR, Aleonard JL, Arditti L, Baranes C, Beaujard J, Beaurain C, Behar M, Beignot-Devalmont P, Biquet D, Blanchard M, Blot E, Bodin X, Bouaniche H, Boulet L, Bourgeois O, Bretillon F, Breton N, Broyer F, Buffler P, Camper E, Carissimo P, Carrera J, Causse P, Cayet JP, Cayron P, Cazard C, Chaix C, Chazerans D, Cheftel JA, Codron G, Cooren G, Coutrey L, Crappier JJ, Daugenet C, Dauzat C, Defreyn F, Delamare G, Delsart D, Demure P, Desmarchelier P, Domenech A, Dubois D Laroy G, Dubrana E, Dumond P, Dumont A, Durel G, Ellé P, Evellin F, Eyraud P, Fhal G, Fournillou JC, Galesne Herceg G, Gastan G, Geoffray B, Giagnorio P, Goguel J, Granger JF, Guenee P, Haushalter B, Huber C, Hureau JP, Jacob L, Jami A, Jordan E, Jourde P, Journet L, Julien D, Jusserand JT, Korsec P, Laforest G, Lalanne G, Le Duff N, Le Guen-Naas A, Le Hir A, Lebois S, Leclerc S, Leclere V, Lejay D, Lejoly JM, Lemoine C, Lepine C, Lepoutre B, Leprince P, Lhoumeau P, Lognos B, Lustig G, Mannessier B, Marlier M, Marmor P, Martocq G, Massot J, Meme B, Mercier P, Mercier G, Mesnier PL, Meyrand G, Mongin G, Montavont J, Morlon P, Pantea D, Parisot J, Partouche H, Pertusa MC, Petot A, Peyrol Y, Piketty B, Poignant G, Pradere H, Rabaud D, Rachine L, Ragon B, Rambaud J, Richard P, Rigaill P, Robinson D, Rosenberg S, Ruetsch M, Sacareau D, Saint Lannes M, Saugues M, Sauvage P, Schaupp T, Schmitt C, Sellam A, Severin JF, Simian B, Specht L, Szmuckler I, Tetaud D, Trehou P, Triantaphylides JC, Triot P, Uge P, Urbain F, Urbina JC, Vailler P, Vallez V, Varnier H, Venot N, Verhun R, Vogel JY, Zanuttini-Vogt C, Zeline V.
